# The Risk of Stroke in Kidney Transplant Recipients with End-Stage Kidney Disease

**DOI:** 10.3390/ijerph16030326

**Published:** 2019-01-24

**Authors:** Shih-Ting Huang, Tung-Min Yu, Ya-Wen Chuang, Mu-Chi Chung, Chen-Yu Wang, Pin-Kuei Fu, Tai-Yuan Ke, Chi-Yuan Li, Cheng-Li Lin, Ming-Ju Wu, Chia-Hung Kao

**Affiliations:** 1 Division of Nephrology, Taichung Veterans General Hospital, Taichung 407, Taiwan; kitheroborn@hotmail.com (S.-T.H.); yu5523@gmail.com (T.-M.Y.); colaladr@yahoo.com.tw (Y.-W.C.); mcchung@vghtc.gov.tw (M.-C.C.); 2 Graduate Institute of Public Health, China Medical University, Taichung 404, Taiwan; 3 Graduate Institute of Biomedical Sciences, School of Medicine, College of Medicine, China Medical University, Taichung 404, Taiwan; 4 Department of Critical Care, Taichung Veterans General Hospital, Taichung 407, Taiwan; chestmen@gmail.com (C.-Y.W.); yetquen@gmail.com (P.-K.F.); 5 Division of Nephrology, Ministry of Health and Welfare Chiayi Hospital, Chiayi 600, Taiwan; nightrider731@msn.com; 6 Graduate Institute of Clinical Medical Science, China Medical University, Taichung 404, Taiwan; cyli168@gmail.com; 7 Department of Anesthesiology, China Medical University Hospital, Taichung 404, Taiwan; 8 Management Office for Health Data, China Medical University Hospital, Taichung 404, Taiwan; orangechengli@gmail.com; 9 College of Medicine, China Medical University, Taichung 404, Taiwan; 10 Department of Nuclear Medicine and PET Center, China Medical University, Taichung 404, Taiwan; 11 Department of Bioinformatics and Medical Engineering, Asia University, Taichung 404, Taiwan

**Keywords:** kidney transplant recipients, stroke, end-stage renal disease, chronic kidney disease

## Abstract

*Background*: The incidence of stroke after kidney transplantation is poorly understood. Our study aimed to determine the incidence and predictors of stroke as well as mortality from stroke in kidney transplant recipients (KTRs). *Methods*: This retrospective cohort study used the National Health Insurance Research Database in Taiwan to study KTRs (*N* = 4635), patients with end-stage renal disease (ESRD; *N* = 69,297), and patients from the general population who were chronic kidney disease (CKD)-free and matched by comorbidities (*N* = 69,297) for the years 2000 through 2010. The risk of stroke was analyzed using univariate and multivariate Cox regression models and compared between study cohorts. *Findings*: Compared with the ESRD subgroup, KTRs had a significantly lower risk of overall stroke (adjusted hazard ratio (aHR) = 0.37, 95% confidence interval (CI) = 0.31–0.44), ischemic stroke (aHR = 0.45, 95% CI = 0.37–0.55), and hemorrhagic stroke (aHR = 0.20, 95% CI = 0.14–0.29). The risk patterns for each type of stroke in the KTR group were not significantly different than those of the CKD-free control subgroup. The predictors of stroke were age and diabetes in KTRs. All forms of stroke after transplantation independently predicted an increased risk of subsequent mortality, and the strongest risk was related to hemorrhagic events. *Interpretation*: KTRs had a lower risk of stroke than ESRD patients, but this risk was not significantly different from that of the CKD-free comorbidities-matched general population group. Although stroke was relatively uncommon among cardiovascular events, it predicted unfavorable outcome in KTRs.

## 1. Introduction

Kidney transplantation (KT) has become the gold standard treatment for patients with end-stage renal disease (ESRD) because of its survival benefit [1,2]. Despite the associated comorbidity burden of kidney transplant recipients (KTRs) in recent years, mortality and major cardiovascular events (CVEs) have steadily declined in KTRs, and become lower than those of a matched chronic kidney disease (CKD) population [3,4]. Even older KTRs have exhibited a significant improvement in survival compared with similar patients on KT waiting lists [5]. The reduction in mortality among KTRs compared with waitlist patients has been attributed in part to a decrease in CVEs [6,7,8,9]. 

However, despite great progress in patient management, KTRs continue to have an increased risk of death and CVEs compared with population controls [10,11,12,13]. The morbidity and mortality from CVEs cannot entirely be accounted for by the conventional CV risk factors such as diabetes, hypertension, and renal dysfunction [6,14,15,16]. Transplant-specific factors such as immunosuppression medications, inflammation, and anemia have also been linked to an increased risk of CVEs in KTRs [17,18]. 

Although KT prevented cardiovascular morbidity and mortality in KTRs [7], the occurrence of major CVEs strongly predicted graft failure and death after transplantation [8,9]. Thus, identifying potentially modifiable risk factors and managing CVEs have become targets for improving outcomes in KTRs [19,20]. 

Stroke, which is among one of the major CVEs, is not as prevalent as other cardiovascular diseases, but contributes to comorbidity and mortality in KTRs. Until recently, little has been known about the epidemiology, risk pattern, and modification of stroke risk after transplantation [17,21,22,23]. Case series concerning KTRs that evaluated stroke risk reported dismal outcomes following stroke [17,21,22]. In these studies, the prevalence of stroke ranged from 3.9–7.9% at various follow-up periods in Western countries, but it was difficult to use comparisons between different demographic regions. Small sample sizes and limited event numbers also limited the generalizability of the results to larger populations of patients. Moreover, these studies were mostly conducted in the early 2000s; thus, an era effect probably influenced the outcomes after advanced management of KTRs. Only one population-based study enrolled controls from a similar set of waitlist patients, and it showed that KT reduced the risk for cerebrovascular disease [23]. However, the risk pattern of stroke in KTRs remains unknown in Asian countries.

We conducted this retrospective study to estimate the absolute and relative risk of stroke among a large cohort of recent KTRs in Taiwan. A comparison of the primary outcome in KTRs to the CKD-free, comorbidities-matched general population and patients with ESRD is made. We also aimed to describe the predictors and mortality implications of stroke in KTRs. 

## 2. Materials and Methods

### 2.1. Data Sources

This retrospective observational cohort study examined data from Taiwan’s Longitudinal Health Insurance Research Database 2000 (LHID2000) and the Registry of Catastrophic Illness Database (RCID), both of which are subsets of the National Health Insurance Research Database (NHIRD), from 1 January 2000 to 31 December 2011. The NHIRD includes all of the beneficiaries’ inpatient and outpatient medical claims and enrolled data from 23 million Taiwanese people (99.9% of the population) in 2015 [24,25]. The LHID2000 was a randomly sampled dataset of 1,000,000 beneficiaries who made claims from 1 January 1998 to 31 December 2009, and was released by the NHIRD [26]. The RCID includes data for all of the patients who fulfilled the guidelines of the National Health Insurance Administration for receiving a catastrophic illness certificate. Catastrophic illnesses are defined as severe medical conditions requiring advanced healthcare such as post-KT status and ESRD. Patients with a catastrophic illness certificate are exempt from copayment for medical care. We used the RCID to identify KTRs and patients with ESRD and LHID2000 to randomly select individuals for the CKD-free healthy control group. The NHIRD identifies diseases using the International Classification of Diseases, Ninth Revision, Clinical Modification (ICD-9-CM). 

### 2.2. Study Design and Participants 

We identified KTRs (ICD-9-CM codes V42.0, 996.81) and patients with ESRD (ICD-9-CM code 585) from the RCID. The index date for KTRs was defined as the date of undergoing KT operation, and the index date for ESRD patients was the first date that the ESRD was diagnosed. We excluded patients younger than 18 years, those with an incident of ESRD within 90 days of the index date, or those who withdrew from the insurance program before the index date. Finally, we enrolled 4635 KTRs for the study cohort and 69,297 patients with ESRD for the comparison cohort. 

For the ESRD group, we selected individuals from the LHID2000 and frequency matched them with the patients with ESRD by age (a span of every five years), sex, hypertension, diabetes, coronary artery disease (CAD), and index year at a 1:1 ratio to create the CKD-free (CKD: ICD-9-CM codes 580–589) and comorbidities-matched healthy control group. To enable comparisons with the KT cohort, we further created two comparison subgroups from the CKD-free healthy controls and the ESRD group to use in subgroup analysis. Individuals in the CKD-free healthy and ESRD subgroups were also frequency matched with the KT cohort by age (every five years), sex, hypertension, diabetes, CAD, and index year at 1:1 and 1:4 ratios. We selected age, diabetes, hypertension, and CAD for the matching criteria to avoid confounding effects, because these were reported to be independent clinical correlates of cerebrovascular events in KTRs [23]. CKD-free healthy control groups served as the control for the age, sex, and comorbidities-matched general population. To ensure the comparability of the comparison cohorts, we used the same exclusion criteria as for the study cohort. 

The follow-up period commenced from the index date until the first occurrence of stroke, which is the date that the patients were censored because of withdrawal from the National Health Insurance program, death, or the end of the study (31 December 2011), whichever occurred first. 

### 2.3. Comorbid Variables and Outcome Measurement: Definitions

Preexisting comorbidities that could have affected the outcome, namely diabetes (ICD-9-CM code 250), hypertension (ICD-9-CM codes 401–405), hyperlipidemia (ICD-9-CM code 272), chronic obstructive pulmonary disease (COPD; ICD-9-CM codes 491, 492, 496), congestive heart failure (CHF; ICD-9-CM code 428), atrial fibrillation (ICD-9-CM code 427.31), and CAD (ICD-9-CM codes 410–414), were investigated. These comorbidities had been diagnosed in previous inpatient claims records or in at least three successive outpatient claims records before the index date, and were used as covariates in multivariable Cox models. 

The primary endpoint was the first diagnosis of stroke in the follow-up period. Stroke was defined by pairing a National Health Insurance claim with the corresponding diagnostic codes (stroke, ICD-9-CM codes 430–438; ischemic stroke, ICD-9-CM codes 433–437; hemorrhagic stroke, ICD-9-CM codes 430–432). In addition to an in-hospital diagnosis, neurologists or clinicians reviewed imaging studies of the brains of patients taken by computed tomography or magnetic resonance imaging within 30 days of stroke onset. The time to the first diagnosis of stroke was computed as the duration from the time of interest (entering KT or ESRD status) to the earliest stroke claim. 

### 2.4. Statistical Analysis

Continuous data are reported as median and interquartile range (IQR). Categorical data are expressed as counts and percentages. The chi-squared test was used to examine the differences between the distributions of demographic statuses and comorbidities in categorical variables between the cohorts. The Mann–Whitney U-test was used to examine the continuous variables. The cumulative incidence of stroke was calculated using the Kaplan–Meier method, and the log-rank test was used to verify the equality of survivor functions between study groups. 

Univariable and multivariable Cox proportional hazards regression models were used to estimate hazard ratios (HRs) and 95% confidence intervals (CIs) to evaluate the risk of stroke. Crude estimations were performed and adjusted for possible confounders. Logistic regression models were used to calculate the odds ratios (ORs) with 95% CIs to evaluate the predictors of disease outcomes. All of the statistical analyses were performed using SAS software, version 9.4 (SAS Institute Inc., Carey, NC, USA). A two-tailed *p* value < 0.05 was considered significant.

### 2.5. Ethics Approval

Patient records were anonymized, and this study was approved for exemption from informed consent rules by the Ethics Review Board of China Medical University and Hospital in Taiwan (CMUH104-REC2-115-CR3).

## 3. Results

### 3.1. Participants

Table 1 displays the demographic and clinical characteristics of the study cohorts. The median ages of patients at the time of entering ESRD and receiving KT were 65.0 and 47.0 years, respectively. The KT group consisted of 52.6% men and the ESRD group was 49.3% men. Comorbidities including COPD, CHF, and a history of stroke were more prevalent in the ESRD subgroup than in the CKD-free control subgroup and KT group. Hypertension (84.9%) and hyperlipidemia (34.3%) were prevalent among KTRs. Prior to transplantation, 21.9% of patients in the KT group had CAD, and 4.53% of patients had had a stroke. Most KT patients received prednisolone, mycophenolate mofetil (MMF), and tacrolimus (99.6%, 85.5%, and 80.0%, respectively) at one year after transplant. The median dialysis time before kidney transplantation was 2.9 years. 

### 3.2. Incidence of Stroke

The incidence density for overall stroke was 5.34, 14.5, and 6.31 events per 1000 person-years in the KT subgroup, ESRD subgroup, and CKD-free control subgroup, respectively (Table 2). The mean follow-up periods for the patients with stroke were 5.89 ± 3.13 years, 5.78 ± 3.13 years, and 5.06 ± 3.36 years in the KT group, CKD-free control subgroup, and ESRD subgroup, respectively. 

Compared with the CKD-free control group, the ESRD group had a significantly higher risk of overall stroke (adjusted HR (aHR) = 2.11, 95% CI = 2.03–2.20), higher risk of ischemic stroke (aHR = 1.84, 95% CI = 1.76–1.93), and a higher risk of hemorrhagic stroke (aHR = 3.38, 95% CI = 3.09–3.69). Compared with the ESRD subgroup, KTRs had a significantly lower risk of overall stroke (aHR = 0.37, 95% CI = 0.31–0.44), ischemic stroke (aHR = 0.45, 95% CI = 0.37–0.55), and hemorrhagic stroke (aHR = 0.20, 95% CI = 0.14–0.29). The risk patterns for each of the stroke types in KTRs were not significantly different from those of the CKD-free control subgroup. 

**Figure 1:**
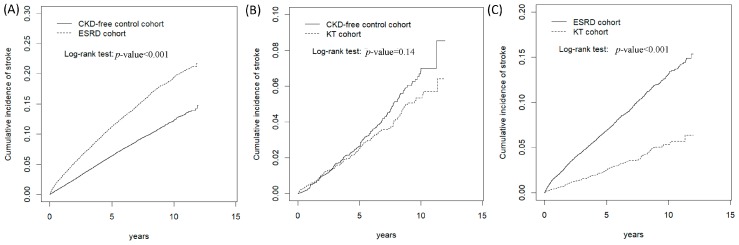
Cumulative incidence curves of stroke in the ESRD and KT groups compared with the CKD-free control group. Cumulative incidence curves of stroke in the ESRD compared with the CKD-free control group (**A**), KT groups compared with the CKD-free control group (**B**), KT groups compared with the ESRD groups (**C**).

Compared with the survival analysis of the CKD-free control subgroup, the ESRD subgroup had a significantly higher cumulative incidence of stroke (log-rank, *p* < 0.0001; Figure 1A). Compared with the ESRD group, KTRs had a significantly lower incidence of stroke (log-rank, *p* < 0.0001; Figure 1C). No significant difference with respect to the cumulative incidence of stroke was evident between the KTRs and the CKD-free control subgroups (Figure 1B). 

### 3.3. Risk of Stroke for the KT, CKD, and ESRD Subgroups Stratified by Age and At-Risk Time

Table 3 presents data indicating that the ESRD group had a significantly higher risk of overall, ischemic, and hemorrhagic stroke compared with the risks for the CKD-free control group among all of the age stratifications, and especially for those aged 20–49, who had the highest overall stroke risk. The KT group had a significantly lower risk of overall, ischemic, and hemorrhagic stroke than the ESRD subgroup among all of the age stratifications, except for the risk of hemorrhagic stroke in KTRs aged above 65 years. The overall and ischemic stroke risks in KTRs were not significantly different compared with the CKD-free control subgroup among all of the age stratifications. We further analyzed the risk of stroke, which was stratified according to at-risk time (less than or more than five years) among the studied cohorts (Table 4), and discovered that the risks of overall, ischemic, and hemorrhagic stroke were higher in the ESRD group than the risks in the CKD-free control group, and that the risks of overall, ischemic, and hemorrhagic stroke were lower in the KT group than those in the ESRD subgroup, regardless of at-risk time. The risks of overall, ischemic, and hemorrhagic stroke in KTRs were similar to the risks in the CKD-free general population, regardless of at-risk time.

### 3.4. Independent Correlates of Stroke after KT

To explore the predisposing factors for stroke in the KT group, we further divided KTRs into patients who experienced stroke and patients who had not, and compared their characteristics. For KTRs with stroke, hypertension was present in 91.8% of patients; meanwhile, 37.7% had hyperlipidemia, and 32.9% reported underlying CAD. The risk of stroke increased significantly with age (adjusted odds ratio (aOR) = 1.02 with 95% CI = 1.01–1.04 for each year of age) and the presence of diabetes (aOR = 2.08 with 95% CI = 1.42–3.03; Table 5). 

### 3.5. Effects of Stroke on Subsequent Mortality after KT

Among the 146 KTRs who had strokes, 61 died during the follow-up period (64.1 events per 1000 person-years) (Table 6). All of the types of CVEs after transplantation independently predicted an increased risk for subsequent mortality, and the strongest risk was associated with hemorrhagic events. Adjusted hazard ratios for death were 4.19 (95% CI 2.72 to 6.43) after hemorrhagic stroke, 1.88 (95% CI 1.20 to 2.34) after ischemic stroke, and 2.30 (95% CI 1.75 to 3.01) for overall stroke in KTRs.

## 4. Discussion

In this population-based study, we determined that the incidence of stroke was not as high as that observed in Western countries. Ischemic stroke was the major subtype of stroke among KTRs. The risk of stroke was significantly lower for KTRs than that for patients with ESRD, but approximated that of the matched general population. The relative risk reduction for stroke among KTRs remained significant after various age and at-risk time stratifications compared with patients with ESRD. Our study was the first study to assess KT in the Asian population with respect to stroke, and our analysis revealed some intriguing findings. 

The overall three-year cumulative incidence of ischemic stroke after KT was 1.05% in our cohort, which was relatively lower than the 3.5% reported in the study of Lentine et al. of Medicare-insured KTRs that examined the United States (U.S.) Renal Data System registry data [23], and approached the 0.7% reported in the study of Lam et al. of KTRs that examined healthcare databases in Ontario, Canada [3]. The higher cumulative incidence of ischemic stroke in the U.S. study compared with our study may be because the U.S. cohort consisted of a higher proportion of older KTRs (22.8% of U.S. recipients were ≥60 years compared with 3.86% of our recipients who were ≥65 years) and patients with diabetes as a comorbid disease (34.4% versus 15.5%). The enrollment era was also different among these countries (1998 through 2002 in the U.S. study, 1997 through 2009 in the Canadian study, and 2000 through 2010 in our study). Similar to the U.S. and Canadian studies, traditional cardiovascular risk factors were highly prevalent in our KTRs as well. 

In Table 1, we could see the comorbid profile was different between the ESRD and the KT group. The proportions of having diabetes, CHF and CAD, were less in the KT group compared with those in the ESRD group, which could be explained by the careful patient selection for transplantation and the improved metabolic profile. The initial maintenance immunosuppressive regimens in the KT group are often triple combinations from glucocorticoids, a calcineurin inhibitor, and an antimetabolic agent. The progress of immunosuppressive regimens, namely from cyclosporine to tacrolimus and from azathioprine to MMF, minimizes the morbidity associated with each class of agent and promotes adherence. The risk of stroke for KTRs approximated that in the matched CKD-free general population despite the different age stratifications and at-risk time in our study (Table 3 and Table 4). This result encourages older patients to benefit from a kidney transplant despite comorbidities, particularly with respect to life quality and life expectancy. 

Until recently, only the study of Lam et al. had compared the risk of CVEs between the general population, patients with CKD, and KTRs, but the assessment of cerebrovascular outcome was limited to ischemic stroke [3]. The author discerned a 6.4% higher risk of death or major CVE in KTRs compared with the general population. Our different results can be attributed to the selection of patients from the CKD-free comorbidities-matched general population for the comparison group. Our findings supported the contention that reducing cardiovascular risk factors is critical for the management of patients after KT as well as the necessity of preserving renal function through CKD management and the modification of immunosuppressants [18,27,28].

KT provides cardiovascular protection against major CVEs; thus, we proposed that the risk of stroke could also be ameliorated by the recovery of renal function. In our study, receiving a KT predicted a 63% risk reduction in overall stroke compared with the ESRD subgroup. This finding resonated with other studies that discerned evidence of a reduced risk for stroke after KT [23]. With respect to the pathogenesis of vascular function after KT, studies from Kensinger et al. reported a significant association between decreased fibroblast growth factor 23 levels and improved left ventricular mass index following transplantation, and that the improved endothelial function after transplantation was maintained for up to two years [29,30]. KT also ameliorated dyslipidemia, which is a well-recognized cardiovascular disease risk factor characterized by a decreased concentration of high-density lipoprotein and an increased concentration of triglycerides that is associated with kidney impairment [31]. Another study demonstrated that arterial stiffness, but not endothelial dysfunction, was associated with multidrug antihypertensive therapy and nondipper blood pressure (BP) in KTRs [32]. However, their study did not evaluate stroke risk in KTRs with respect to optimal or suboptimal BP control. 

Since donor characteristics were unavailable from the RCID, we used the statistics of kidney transplantation from the Taiwan Organ Registry and Sharing Center (TORSC), which is an organ sharing and registration network funded by the Department of Health since 2002. The ratio of cadaveric transplantation to living transplantation was approximately 2:1 [33]. To compare transplantation outcomes in Taiwan with other countries, Chiang et al. reviewed data from the TORSC from 2005 to 2013, which demonstrated that the nationwide three-year survival rate after KT was 93.72% for patients and 83.08% for kidney grafts, which was not inferior to the findings in the Organ Procurement and Transplantation Network/Scientific Registry of Transplant Recipients from 2008 [34]. Among the deceased donation in 2012, 91.9% of donors were standard criteria donors, and 8.9% donors were expended criteria donors [34]. In this study, all of the types of stroke had adverse mortality implications and the outcome was poor, as 41% (61/146) of stroke patients died during the follow-up period. Our findings corresponded to the result that nearly half of the KTRs died in the three months following stroke [21].

In our study, the main predictors of stroke in KTRs were diabetes and age, which accorded with the results of other studies [21,22,23]. The comorbid conditions of hypertension and CAD were significant risk factors in the univariate analysis, but their effects were reduced in the multivariate analysis, which was perhaps because of the large influence of age and diabetes. The strong association between age and stroke risk in KTRs may indicate a need to address cardiovascular risk more aggressively in older potential KTRs. 

Our study was strengthened by its large sample size, the long duration of follow-up, and our estimates of excess risk being adjusted not only for age, sex, and index date, but also comorbidities. Despite these strengths, the results of this study must be viewed in light of the following limitations. This was a retrospective observational study, and unpredictable confounding factors may have influenced the outcomes. To overcome this limitation, we used frequency matching to balance the confounding factors among the cohorts, thereby mitigating the inherent difference at baseline. Second, we relied on administrative data concerning smoking, donor characteristics (donor sex, traits, and comorbidities), and certain transplantation factors (sensitized recipient, human leukocyte antigen (HLA) match, delayed graft function, and biochemical laboratory data) that have been helpful but were not available in our database. However, our database has been validated with major diseases [35,36]. Third, the lower prevalence of diabetes due to a selection has been justified by the careful patient selection for transplantation and the improved metabolic profile. This implies a lower vascular risk in the transplanted patients than in the controls. Although it may have little impact on the overall results, this lower risk in the discussion should reflect that patients have a slightly lower risk due to selection bias. Finally, because our study included predominantly Asian patients, the results may not be generalizable to other ethnic populations. 

## 5. Conclusions

We discovered that the risk for all types of stroke was lower among KTRs than patients with ESRD, and was approximate to that of the general population. We also concluded that stroke was relatively uncommon after KT, but it predicted an increased risk for death in KTRs. We believe that vigilance in detecting and controlling modifiable CVE risk factors may be critical for reducing the risk of stroke and eventual death among KTRs.

## Figures and Tables

**Table 1:** Demographic characteristics and comorbidities of study participants according to disease status.

Baseline Characteristics	^‡^ CKD-Free Control *N* = 69,297		ESRD *N* = 69,297		ESRD vs. ^‡^ CKD-Free Control *p*-Value	^ǂ^ CKD-Free Control Subgroup *N* = 4635		^ǂ^ ESRD Subgroup *N* = 18,540		KT *N* = 4635		KT vs. ^ǂ^ CKD-Free Control Subgroup *p*-Value	KT vs. ^ǂ^ ESRD Subgroup *p*-Value
Variable	*N*	%	*N*	%		*N*	%	*N*	%	*N*	%		
**Age, years**					0.99							0.99	0.99
20–49	13,658	19.7	13,658	19.7		2787	60.1	11,148	60.1	2787	60.1		
50–64	20,968	30.3	20,968	30.3		1669	36.0	6676	36.0	1669	36.0		
≥65	34,671	50.0	34,671	50.0		179	3.86	716	3.86	179	3.86		
Median (IQR) ^&^	65.1 (52.8, 74.8)		65.0 (52.7, 74.7)		0.75	47.3 (38.2, 54.4)		47.8 (40.8, 56.3)		47.0 (38.2, 54.1)		0.22	0.001
**Sex**					0.99							0.99	0.98
Female	35139	50.7	35139	50.7		2198	47.4	8796	47.4	2198	47.4		
Male	34158	49.3	34158	49.3		2437	52.6	9744	52.6	2437	52.6		
**Comorbidity**													
Hypertension	60,325	87.1	60,325	87.1	0.99	3937	84.9	15,674	84.5	3937	84.9	0.99	0.50
Diabetes	21,158	30.5	21,158	30.5	0.99	719	15.5	2876	15.5	719	15.5	0.99	0.99
Hyperlipidemia	27,828	40.2	27,152	39.2	<0.001	1527	32.9	5887	31.8	1588	34.3	0.18	0.001
COPD	3112	4.49	13,451	19.4	<0.001	53	1.14	1636	8.82	345	7.44	<0.001	0.003
CHF	6476	9.35	18,210	26.3	<0.001	159	3.43	3015	16.3	550	11.9	<0.001	<0.001
Atrial fibrillation	1671	2.41	1240	1.79	<0.001	27	0.58	310	1.67	112	2.42	<0.001	0.001
CAD	26,180	37.8	26,180	37.8	0.99	1016	21.9	4038	21.8	1016	21.9	0.99	0.84
History of Stroke	6701	9.67	12,325	17.8	<0.001	167	3.60	1756	9.47	210	4.53	0.02	<0.001
**Immunosuppressive medications, 1-year**													
Prednisolone										4617	99.6		
Cyclosporine										2478	53.5		
Azathioprine										485	10.5		
Tacrolimus										3708	80.0		
Sirolimus										2089	45.1		
Everolimus										383	8.26		
Mycophenolate mofetil or mycophenolate sodium										3964	85.5		
**Dialysis time before KT**, Median (IQR)										2.90 (0.98, 5.20)			

Abbreviations: SD, standard deviation; CAD, coronary artery disease; CHF, congestive heart failure; COPD, chronic obstructive pulmonary disease; KT, kidney transplantation; IQR, interquartile range; ^&^: Mann-Whitney U-test; ^‡^: CKD-free healthy controls were frequency matched with the ESRD group by age, sex, hypertension, diabetes, CAD, and index-year at a 1:1 ratio. ^ǂ^: Two comparison subgroups from the CKD-free healthy controls and ESRD groups were frequency matched with the KT cohort by age, sex, hypertension, diabetes, CAD, and index-year at 1:1 and 1:4 ratios.

**Table 2:** Crude and adjusted hazard ratios for stroke in the ESRD, KT, and control groups.

Outcome variable	ESRD vs. ^‡^ CKD-Free Control		KT vs. ^ǂ^ CKD-Free Control Subgroup KT vs. ^ǂ^ ESRD Subgroup		
	^‡^ CKD-Free Control *N* = 69,297	ESRD *N* = 69,297	^ǂ^ CKD-Free Control Subgroup *N* = 4635	ESRD Subgroup *N* = 18,540	KT *N* = 4635
**Mean follow-up duration, years**	5.05 ± 3.06	3.65 ± 3.09	5.78 ± 3.13	5.06 ± 3.36	5.89 ± 3.13
**No. of Stroke**	4588	6013	169	1356	146
Person-years	350,084	253,210	26,803	93,775	27,319
Incidence rates	13.1	23.8	6.31	14.5	5.34
Adjusted HR (95% CI) ^†^	1.00	2.11 (2.03, 2.20) ***	1.00		0.86 (0.68, 1.08)
Adjusted HR (95% CI) ^†^				1.00	0.36 (0.30, 0.42) ***
**No. of Ischemic stroke**	3894	4344	133	813	112
Incidence rates	11.1	17.1	4.96	8.67	4.10
Adjusted HR (95% CI) ^‡^	1.00	1.84 (1.76, 1.93) ***	1.00		0.85 (0.66, 1.11)
Adjusted HR (95% CI) ^‡^				1.00	0.45 (0.37, 0.55) ***
**No. of Hemorrhagic stroke**	748	1706	38	554	33
Incidence rates	2.14	6.74	1.42	5.91	1.21
Adjusted HR (95% CI) ^‡^	1.00	3.38 (3.09, 3.69) ***	1.00		0.78 (0.48, 1.27)
Adjusted HR (95% CI) ^‡^				1.00	0.20 (0.14, 0.29) ***

Abbreviations: IR, incidence density rates per 1000 person-years; HR, hazard ratio; CI, confidence interval; ^‡^: CKD-free healthy controls were frequency matched with the ESRD group by age, sex, hypertension, diabetes, CAD, and index-year at a 1:1 ratio. ^ǂ^: Two comparison subgroups from the CKD-free healthy controls and ESRD groups were frequency matched with the KT cohort by age, sex, hypertension, diabetes, CAD, and index year at 1:1 and 1:4 ratios. ^†^ Adjusted for age, sex, and the comorbidities of hypertension, diabetes, hyperlipidemia, COPD, congestive heart failure, atrial fibrillation, CAD, and a history of stroke. *** *p* < 0.001.

**Table 3:** Adjusted hazard ratios for stroke among the ESRD, KT, and CKD-free control groups stratified by age.

Age Stratification	ESRD Compared to ^‡^ CKD-Free Control	KT Compared to ^ǂ^ CKD-Free Control Subgroup	KT Compared to ^ǂ^ ESRD Subgroup
	Adjusted HR (95% CI)	Adjusted HR (95% CI)	Adjusted HR (95% CI)
**Stroke**			
Model 1 ^†^			
20–49	3.15 (2.77, 3.58) ***	0.81 (0.58, 1.13)	
50–64	2.46 (2.28, 2.66) ***	0.93 (0.66, 1.31)	
≥65	1.70 (1.61, 1.80) ***	0.72 (0.32, 1.66)	
Model 2 ^†^			
20–49			0.72 (0.57, 0.91) **
50–64			0.66 (0.55, 0.80) ***
≥65			0.35 (0.17, 0.71) ***
**Ischemic stroke**			
Model 1 ^†^			
20–49	2.57 (2.19, 3.01) ***	0.91 (0.61, 1.34)	
50–64	1.97 (1.80, 2.16) ***	0.83 (0.56, 1.22)	
≥65	1.58 (1.49, 1.67) ***	0.71 (0.31, 1.65)	
Model 2 ^†^			
20–49			0.48 (0.36, 0.64) ***
50–64			0.38 (0.28, 0.52) ***
≥65			0.37 (0.19, 0.75) **
**Hemorrhagic stroke**			
Model 1 ^†^			
20–49	4.24 (3.42, 5.24) ***	0.49 (0.26, 0.96) *	
50–64	4.58 (3.87, 5.40) ***	1.36 (0.63, 2.93)	
≥65	2.39 (2.10, 2.72) ***	-	
Model 2 ^†^			
20–49			0.17 (0.10, 0.28) ***
50–64			0.24 (0.15, 0.41) ***
≥65			0.23 (0.03, 1.81)

Abbreviations: HR, hazard ratio; CI, confidence interval; Model 1 and Model 2 ^†^: adjusted for age, sex, and the comorbidities of hypertension, ^‡^: CKD-free healthy controls were frequency matched with the ESRD group by age, sex, hypertension, diabetes, CAD, and index-year at a 1:1 ratio. ^ǂ^: Two comparison subgroups from the CKD-free healthy controls and ESRD groups were frequency matched with the KT cohort by age, sex, hypertension, diabetes, CAD, and index-year at 1:1 and 1:4 ratios. Model 1: The regression model was used to make comparisons between the ESRD and ^‡^ CKD-free control groups and KT and ^ǂ^ CKD-free control subgroups; Model 2: The regression model was used to compare the KT and ^ǂ^ ESRD subgroups diabetes, hyperlipidemia, COPD, congestive heart failure, atrial fibrillation, CAD, and a history of stroke. * *p* < 0.05, ** *p* < 0.01, *** *p* < 0.001.

**Table 4:** Adjusted hazard ratios for stroke in the ESRD, KT, and control groups stratified by at-risk time.

At Risk Time	ESRD vs. ^‡^ CKD-Free Control		KT vs. ^ǂ^ CKD-Free Control Subgroup KT vs. ^ǂ^ ESRD Subgroup		
	^‡^ CKD-Free Control *N* = 69,297	ESRD *N* = 69,297	^ǂ^ CKD-Free Control Subgroup *N* = 4635	^ǂ^ ESRD Subgroup *N* = 18,540	KT *N* = 4635
	Adjusted HR (95% CI)	Adjusted HR (95% CI)	Adjusted HR (95% CI)	Adjusted HR (95% CI)	Adjusted HR (95% CI)
**Stroke**					
Model 1 ^†^					
≤5	1.00	2.15 (2.05, 2.25) ***	1.00		0.97 (0.73, 1.30)
>5	1.00	1.99 (1.82, 2.17) ***	1.00		0.70 (0.48, 1.01)
Model 2 ^†^					
≤5				1.00	0.34 (0.27, 0.42) ***
>5				1.00	0.40 (0.29, 0.53) ***
**Ischemic stroke**					
Model 1 ^†^					
≤5	1.00	1.86 (1.76, 1.96) ***	1.00		0.95 (0.68, 1.31)
>5	1.00	1.79 (1.62, 1.99) ***	1.00		0.72 (0.47, 1.10)
Model 2 ^†^					
≤5				1.00	0.45 (0.35, 0.57) ***
>5				1.00	0.46 (0.33, 0.65) ***
**Hemorrhagic stroke**					
Model 1 ^†^					
≤5	1.00	3.54 (3.20, 3.93) ***	1.00		0.97 (0.51, 1.85)
>5	1.00	2.86 (2.36, 3.46) ***	1.00		0.57 (0.27, 1.20)
Model 2 ^†^					
≤5				1.00	0.17 (0.11, 0.26) ***
>5				1.00	0.29 (0.16, 0.51) ***

Abbreviations: HR, hazard ratio; CI, confidence interval; ^‡^: CKD-free healthy controls were frequency matched with the ESRD group by age, sex, hypertension, diabetes, CAD, and index-year at a 1:1 ratio. ^ǂ^: Two comparison subgroups from the CKD-free healthy controls and ESRD groups were frequency matched with the KT cohort by age, sex, hypertension, diabetes, CAD, and index-year at 1:1 and 1:4 ratios. Model 1: The regression model was used to make comparisons between the ESRD and **^‡^** CKD-free control groups and KT and ^ǂ^ CKD-free control subgroups; Model 2: The regression model was used to compare the KT and ^ǂ^ ESRD subgroups; Model 1 and Model 2 ^†^: Adjusted for age, sex, and the comorbidities of hypertension, diabetes, hyperlipidemia, COPD, congestive heart failure, atrial fibrillation, CAD, and a history of stroke. *** *p* < 0.001.

**Table 5:** Descriptive characteristics of KTRs with and without stroke and odds ratios and 95% confidence intervals for stroke associated with descriptive characteristics.

Variable	Non-stroke KTRs *N* = 4489		Stroke KTRs *N* = 146		Crude OR (95% CI)	^‡^ Adjusted OR (95% CI)
Characteristics	*N*	(%)	*N*	(%)		
**Age, years**						
20–49	2718	60.6	69	47.3	1.00	
50–64	1603	35.7	66	45.2	1.62 (1.15, 2.29) ***	
≥65	168	3.74	11	7.53	2.58 (1.34, 4.97) ***	
Means (SD)	45.9	11.4	50.2	10.9	1.04 (1.02, 1.05) ***	1.02 (1.01, 1.04) **
**Sex**						
Female	2137	47.6	61	41.8	1.00	
Male	2352	52.4	85	58.2	1.27 (0.91, 1.77)	
**Comorbidity**						
Hypertension	3803	84.7	134	91.8	2.01 (1.11, 3.66) ***	1.52 (0.83, 2.79)
Diabetes	672	15.0	47	32.2	2.70 (1.89, 3.85) ***	2.08 (1.42, 3.03) ***
Hyperlipidemia	1533	34.2	55	37.7	1.17 (0.83, 1.64)	-
COPD	331	7.37	14	9.59	1.33 (0.76, 2.34)	-
Congestive heart failure	526	11.7	24	16.4	1.48 (0.95, 2.32)	-
Atrial fibrillation	111	2.47	1	0.68	0.27 (0.04, 1.96)	-
CAD	968	21.6	48	32.9	1.78 (1.25, 2.54) ***	1.24 (0.85, 1.80)
History of Stroke	210	4.68	0	0.00	-	-
**Dialysis time before kidney transplantation**, means (SD)	3.45	3.26	3.03	2.63	0.95 (0.90, 1.01)	-

Data are presented as the number of patients in each group, and percentages are given in parentheses. ^‡^: Adjusted for age, hypertension, diabetes, and CAD. ** *p* < 0.01; *** *p* < 0.001.

**Table 6:** Overall death and hazard ratio of death measured for stroke patients among the KT group.

Title	Stroke	
	No	Yes
	(*N* = 4489)	(*N* = 146)
Person-years	26,742	952
Death	561	61
IR ^a^	21.0	64.1
Adjusted HR (95% CI) ^‡^	1 (Reference)	2.30 (1.75, 3.01) ***
Hemorrhagic stroke		
Adjusted HR (95% CI) ^‡^	1 (Reference)	4.19 (2.72, 6.43) ***
Ischemic stroke		
Adjusted HR (95% CI) ^‡^	1 (Reference)	1.88 (1.20, 2.34) ***

Abbreviations: IR ^a^, incidence density rates per 1000 person-years; HR, hazard ratio; CI, confidence interval; ^‡^: Adjusted for age, hypertension, diabetes, and CAD. *** *p* < 0.001.
